# Exosomes: an overview of biogenesis, composition and role in ovarian cancer

**DOI:** 10.1186/1757-2215-7-14

**Published:** 2014-01-25

**Authors:** Allison Beach, Huang-Ge Zhang, Mariusz Z Ratajczak, Sham S Kakar

**Affiliations:** 1 Department of Physiology and Biophysics, James Graham Brown Cancer Center, University of Louisville, 505 South Hancock Street, Louisville, KY 40202, USA; 2 Department of Microbiology and Immunology, James Graham Brown Cancer Center, University of Louisville, Louisville, KY, USA; 3 Department of Medicine, James Graham Brown Cancer Center, University of Louisville, Louisville, KY, USA

**Keywords:** Exosome, Ovarian cancer, Cancer, Microvesicle, Cancer diagnosis

## Abstract

Exosomes are tiny membrane-bound vesicles that are over produced by most proliferating cell types during normal and pathological states. Their levels are up-regulated during pregnancy and disease states such as cancer. Exosomes contain a wide variety of proteins, lipids, RNAs, non-transcribed RNAs, microRNAs and small RNAs that are representative to their cellular origin and shuttle from a donor cell to a recipient cell. From intercellular communication to tumor proliferation, exosomes carry out a diverse range of functions, both helpful and harmful. Useful as biomarkers, exosomes may be applicable in diagnostic assessments as well as cell-free anti-tumor vaccines. Exosomes of ovarian cancer contain different set of proteins and miRNAs compared to exosomes of normal, cancer-free individuals. These molecules may be used as multiple “barcode” for the development of a diagnostic tool for early detection of ovarian cancer.

## Introduction

Exosomes, like the intraluminal vesicles range approximately from 30-100 nm in diameter secreted by live cells and were first observed in the early 1980s. Secretion of exosomes from cells was proposed to be a mechanism through which cells discard their garbage [1-4]. However, in recent years, exosomes have emerged as important molecules for inter-cellular communication that are involved both in normal and in pathophysiological conditions, such as lactation [5], immune response [6] and neuronal function [5], and also in the development and progression of diseases such as liver disease [7], neurodegenerative diseases [8] and cancer [9,10]. They exhibit a round, saucer-like morphology and are encapsulated by a lipid bilayer [10,11]. Exosomes are found in higher concentrations in the peripheral circulation during pregnancy and cancer [12]. A large number of biological functions of exosomes have been demonstrated however, in this review we will focus discussing on the functions of exosomes in ovarian cancer and immune tolerance related to cancer.

### Exosome biogenesis and secretion

Exosomes in eukaryotic cells, throughout their life cycle periodically engulf small amounts of intracellular fluid, forming a small intracellular body called an endosome [13,14]. As the early endosome matures and develops into the late endosome, it becomes characterized by the formation of intraluminal vesicles (ILV) inside the lumen of the endosome. Ranging from 30-100 nm in diameter, the ILVs are formed by inward budding of the endosomal membrane, randomly engulfing portions of the cytosolic contents and incorporating transmembrane and peripheral proteins into the invaginating membrane. The transformation into the late endosome can also be detected by observing a change in its shape and location: the early endosome is tube-like in shape and is usually located in the outer portion of the cytoplasm, whereas, the late endosome is spherical and found closer to the nucleus [14]. The late endosome containing ILVs is also called as multivesicular body (MVB) [15,16].

The fate of the MVB may vary. Typically, it fuses with the lysosome and its contents are degraded by the hydrolysis inside the lysosome. It has been proposed that the contents of the MVB are sorted; proteins destined for degradation by the lysosome and are found in the ILVs, whereas proteins that may have another function or role are found outside the ILVs of the MVB. However, the mechanism by which this process occurs is not yet completely understood. Possible theories involving the sorting of proteins have been proposed [17,18].

**Figure 1:**
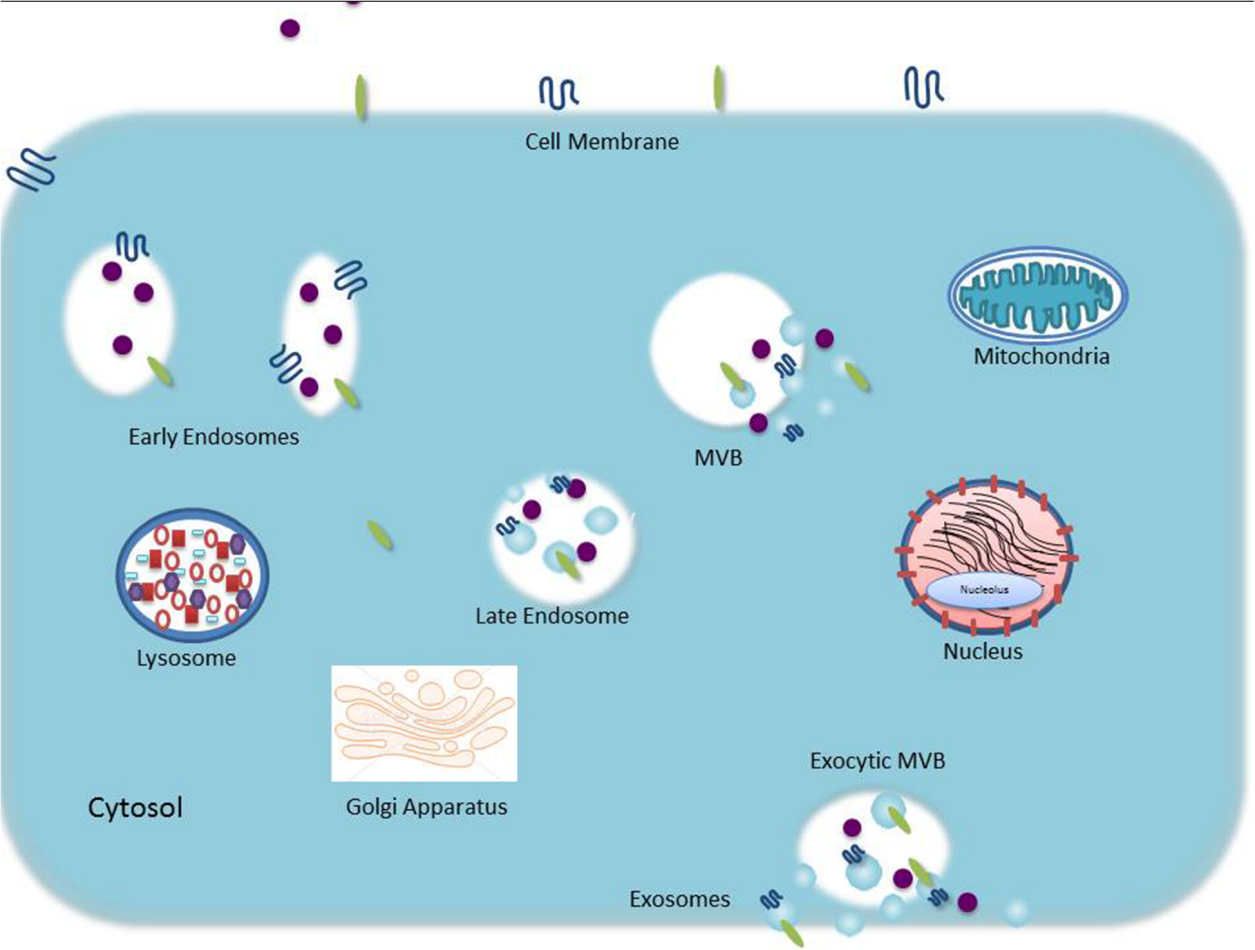
Biogenesis and release of exosomes: diagram depicted the well-accepted model for exosome biogenesis and release.

As an alternative to fusion with the lysosome, the MVB may fuse with the plasma membrane of the cell in a proposed ion-dependent manner instead, releasing its ILVs in an exocytotic fashion to the extracellular environment (Figure 1). These vesicles are then referred as exosomes [15]. Demonstrated both *in vivo* and *in vitro*, exosomes are secreted during both normal and pathological conditions and by a wide array of cell types [19]. First discovered in maturing mammalian reticulocyte (immature red blood cells) exosomes are secreted by wide range of mammalian cell types, including B and T cells [20], dendritic cells [16], mesenchymal stem cells [21], epithelial cells [22,23], astrocytes [24], endothelial cells [25] and cancer cells [12,26-37]. Exosomes have also been identified in most bodily fluids including urine and amniotic fluid [38], blood [39], serum [12,40], saliva [41,42], ascites [28,43], breast milk [6], cerebrospinal fluid [44,45] and nasal secretion [46]. Importantly, cancer cells have been shown to secrete exosomes in greater amounts than normal cells [12,47].

Exosomes are mainly secreted by two different mechanisms, constitutive release via the Trans-Golgi network and inducible release [48,49]. Studies from Thery and colleagues demonstrated that a number of Rab family proteins, including Rab27a and Rab27b, act as key regulators of exosomes secretion [50]. Apart from Rab 27a and 27b, another Rab family member, Rab 35 and Rab 11 have also been shown to regulate the secretion of exosomes by interacting with GTPase-activating protein TBC1 domain family member 10A-C (TBC1D10A-C) [51,52]. The Rab family of proteins are often mutated (constitutively active) or over-expressed in cancer cells.

**Figure 2:**
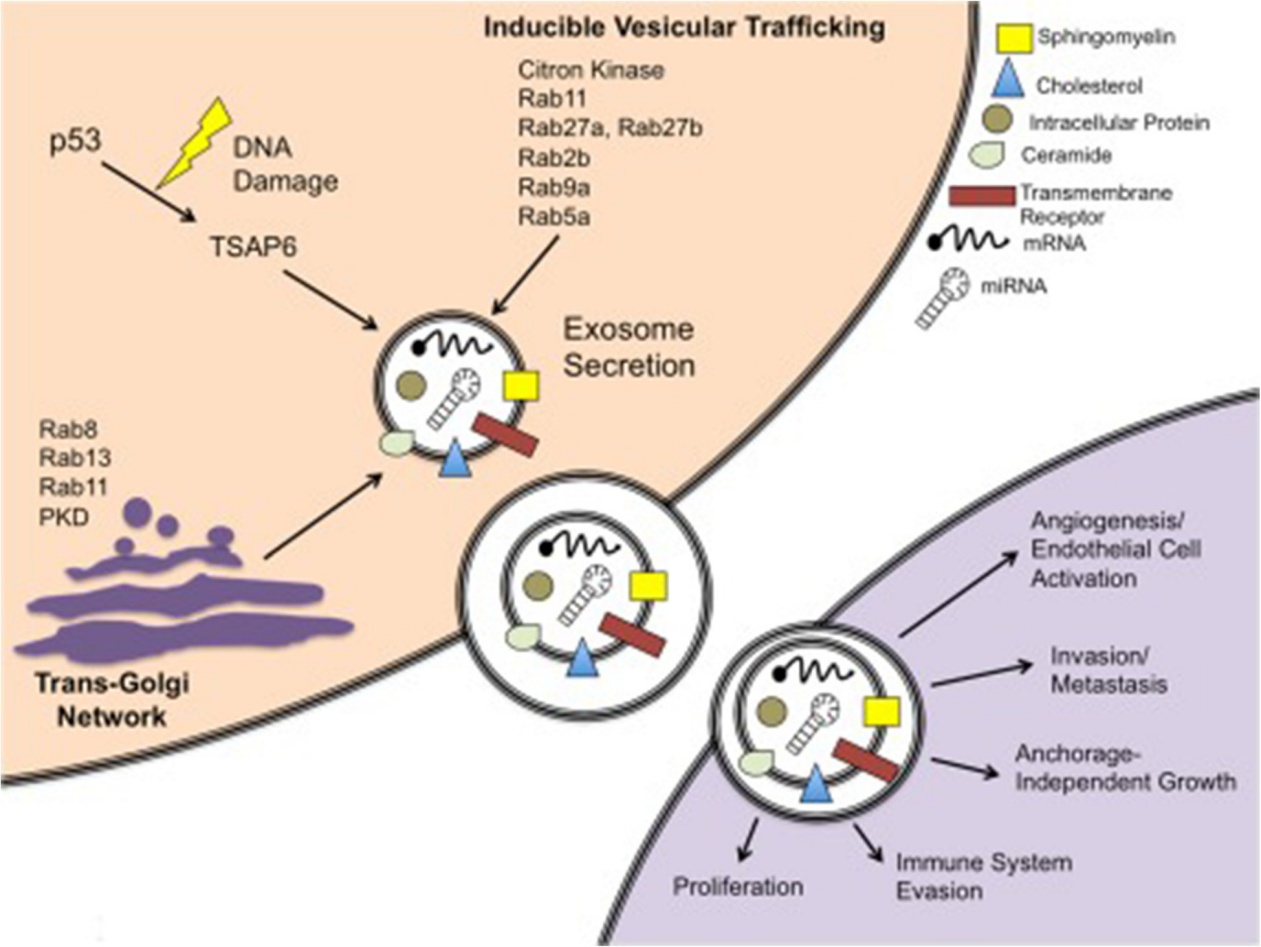
**Schematic of exosome secretion in a cancer cell model.** Exosomes are secreted via a constitutive pathway involving the Trans-Golgi Network and/or inducible pathways, which can be activated by p53 and other stimuli such as Rab proteins. Reproduced from Henderson and Azorsa (19).

It has also been shown that activation of the tumor suppressor protein, p53, stimulates and increases the rate of exosome secretion by regulating transcription of the various genes such as TSAP6 and CHMP4C, which activate exosome production [53]. If the cell experiences stresses such as hypoxia or toxic stress, DNA may become damaged. The p53 protein responds to this process by regulating the transcription of number of genes (Figure 2). This results in what is known as “bystander effect”, during which the cell secretes certain proteins that communicate with nearby cells to help in recruiting them for a response to compensate the stress [54]. Irradiation of human prostate cancer cells strongly indicated DNA damage that could indeed induce a p53-dependent increase in exosome secretion by activating the cells [54]. In addition, secretion of exosomes appears to be increased by K^+^-induced depolarization in neuronal cells, and the crosslinking of CD3 with T cells, and intracellular Ca^2+^ levels [14,15]. Various other stimuli and change in membrane pH have also been shown to trigger the secretion of exosomes from various cell types [55,56].

Once exosomes are released from the parent cells, they carry out a variety of functions and may eventually be taken up by another cell [57] (Figure 2). It has been reported that certain exosomes, such as those that have been secreted by tumor cells, carry phosphatidylserine (PS) on their membranes. PS acts as a signal and has a large role in exosome uptake by certain cells [58]. When an exosome is taken up by another cell, the entire exosome may be endocytosed and re-legated to clathrin-coated pits, or may simply release its inner contents into the cell and remain united with the cell membrane [57].

### Composition of exosomes

A large amount of exosomal proteins have been identified, and the consistency among exosomes stemming from like-cell types suggests that a sorting mechanism is acting during exosomal development. TSG101 and Alix have been identified in exosomal analysis and are known components of the *endosomal sorting complex required for transport* (ESCRT) machinery. ESCRT functions to sort the cargo proteins of ILVs. It works at the endosomal/MVB membrane and recognizes ubiquitinylated proteins that are associated with tetraspanins and possibly those which are GPI-anchored, arranges them in such a way on the membrane that they are fated to become contents of exosomes [15,17].

The function of the original cell from which an exosome originate can be deduced by the makeup of proteins, lipids, miRNAs, mRNA, non-coding RNAs and other molecules found in a particular exosome. Exosomes contain many different cell surface molecules and are able to engage many different cell receptors simultaneously. This allows them to participate in the exchange of materials between cells, for example proteins, lipids, carbohydrates, and pathogens [13]. Recent information from different cell type reveals that exosomes contains 4,563 proteins, 194 lipids, 1639 mRNA and 764 microRNA [59,60] demonstrating their complexity. Exosomes are rich in molecules that are involved in antigen presentation, such as CD1 and the major histocompatibility molecules (MHC) class I and II, which play a key role in immune-regulation by possessing antigenic peptides [61]. MHC I also may be involved in the presentation of antigen fragments to T cells [62,63]. Also exosomes contain tetraspanins CD9, CD63, CD81, and CD82, as well as co-stimulatory molecules (CD86) and adhesion molecules CD11b and CD54 [33,61,62]. Other common exosomal proteins include heat shock proteins HSP70 and HSP90, which expedite peptide loading onto MHC I and II, as well as play a role in the cellular response to environmental stresses. Inside the cell, HSPs assist in protein folding and trafficking, acting as chaperones [62,63]. Exosomes in general also contain cytoplasmic proteins such as annexins and rab proteins, both of which may promote the fusion of MVB with the cell membrane and expulsion of exosomes. In the last few years, exosomes have been reported to contain nucleic acid such as DNA, RNA, miRNA and none coding RNA [12,58,59,64-69] in addition to proteins. RNA containing exosomes may represent alternate pathway of cellular communication with significant implications in the modification of cell phenotypes. The mRNAs found in exosomes are found to be functional and could be transferred to target cells and translated into proteins [70,71]. Specific miRNA such as let-7, miR-1, miR-15, miR-16, miR-151 and miR 375 which play role in angiogenesis, hematopoiesis, exocytosis and tumorigenesis have been reported in exosomes [65]. Along with DNA, RNA, miRNA, cytoplasmic tubulin, actin, and actin-binding proteins are also commonly found in exosomes [12,58,59]. Membrane proteins CD55 (decay accelerating factor) and CD59 have been reported in exosomes which provide protection against the complement system and thus are an instrumental part in the stability of the exosome outside the cell [15]. Exosomes also commonly contain signal transduction proteins such as G-proteins and protein kinases [58,59]. In addition, exosomes tend to have a rich content of proteolysis enzymes, suggesting that exosomes may increase cell migration [10,13]. It is to note that exosomes do not contain mitochondrial, nuclear, and endoplasmic proteins [13].

Exosomes derived from B cells, T cells, dendritic cells, intestinal epithelial cells and many other cell types contain specific-proteins pertaining to their particular functions in addition to commonly found proteins in exosomes. Exosomes secreted by intestinal epithelial cells display syntaxin 3, C26, and the A33 antigen, depending upon which side of the epithelial membrane it is released such as apical or basolateral [15]. Exosomes from T lymphocytes contain many T cell receptors, indicating exosomes role in intercellular communication. Exosomes secreted by dendritic cells contain CD80 and CD86 proteins, which are involved in the activation of naïve CD4^+^ T cells [10]. Microglial cells appear to release exosomes, which display CD13, the active aminopeptidase that cleaves neuropeptides [15]. Some of the major proteins reported in the exosomes are shown in Table 1.

**Table 1:** Some of the common proteins in exosomes

**Antigen presentation**	**MVB formation**	**Signal transduction**	**Heat shock**	**Tetraspanin**	**Adhesion molecules**
MHC I	Alix	Gi2a, Gi3a, Gsa, FRL, Erk2, Fyn	Hsp70	CD9	CD146, CD166
MHC II	TSG101		Hsp84	CD37	ICAM-1, ALCAM
CD86	Gag	Sh2 phosphatase	Hsp90	CD53	MAC-1, Integrins a3, a4, aM, b1, b2
		RhoA, C, GDI		CD63	
		Syntenin, CBL		CD81	LFA3, CD53,
		Catenin, LCK		CD82	CD326
					CD11a, b, c
**Membrane**	**Cytoskeletal**	**Miscellaneous**		**Lipid Raft**	**Enzymes**
**Transport & Fusion**	**Proteins**			**Associated**	
AP-1, Arp2/3, SNAP	Radixin	Thioredoxine	P-Selectin	LBPA	ATP Citrate lyase
Syntaxin	Advillin	peroxidase	CD18, CD147	Cholesterol	ATPase
			Clathrin		
Dynamin	Vimentin	Histone 1, 2, 3	Complement factor 3	Flotillin-1	G6P Isomerase
Rab5,7, Rap1B	Talin, CAP1	MVP, CD26, CD13	CD55	Stomatin	Peroxiredoxin 1
RabGDI	Ezrin, Actin		CD59		
					Asp amino- transferase
					a-enolase
Annexins (I, II, IV, V, VI)	Tubulin	Ferritin light chain 1, 2			Aldehyde reductase
	Moesin				FA Synthase
	Cofilin				Pyruvate kinase
References:	15, 6, 72,73				

### Functions of exosomes

Exosomes are involved in many physiological processes, both beneficial and pathological. Initially, exosomes were characterized as a means for the maturing reticulocyte to get rid of superfluous proteins. Studies have now shown that exosomes are involved in elimination of unnecessary proteins or unwanted molecules from the cell, exchange of materials between cells, intercellular communication, propagation of pathogens, functions of the immune system (both stimulatory and inhibitory), antigen presentation, and many more. By observing the varying protein contents, it is quite easy to understand how exosomes that come from different cell types can have differential functions [13,62] and prove to be highly useful molecules in various biological functions.

### Exosomes in ovarian tumor

There is a paradox about exosomes that some may have beneficial effects, such as those released from B cells and dendritic cells. These have powerful anti-tumor and immune-stimulatory effects and the latter also provides co-stimulatory proteins that are used to activate T cells when presented with tumor antigens. On the other hand, exosomes secreted from tumor cells have been observed to enhance tumor invasiveness and angiogenesis, while suppressing the immune response [72]. Breast cancer tumor-derived exosomes and prostasomes (exosome-like vesicles released by the prostate) can also promote angiogenesis in individuals with cancer, whereas prostasomes in a healthy individual inhibit angiogenesis, enhance sperm motility, and delay and stabilize the acrosome reaction, as well as act immune suppressive in its local environment [35,57]. It is not only cancer cell-derived exosomes that have pervasive qualities; exosomes secreted by platelets have demonstrated to promote tumor growth and metastasis of lung cancer cells [55].

Tumor cell-secreted exosomes include many of the common exosomal proteins; they also contain tumor antigens that are reflective of the tumors they are derived from [13]. The heat shock proteins (HSP) found in tumor exosomes are taken up by dendritic cells and macrophages, which process and present the HSP to the lymph nodes. The HSP then release the tumor-specific antigens they hold, which are presented to cytotoxic T cells leading to their activation. Studies have demonstrated the elimination of various cancers using HSP-stimulated immune cells [62].

Exosomes released by tumor cells are quite unique and have recently come into the spotlight, with many groundbreaking discoveries of their unique proteins and their potential to be diagnostic biomarkers. Tumor exosomes are known to have a significant role in the communication and interaction with tumor cells, immune cells and its surrounding environment. In cancer cells, exosomes entail the transfer of cancer-promoting cellular contents to surrounding cells within the tumor microenvironment or to the circulation to act at distant sites, thereby enabling cancer progression. Several studies have demonstrated the presence of exosomes in ovarian cancer cell cultures and ovarian cancer patients’ plasma/serum or ascites [12,38,39,43,58,62,63,73-76] in addition to many other cancers. Three to 4-fold higher amount of exosomes has been reported in circulation of patients with ovarian cancer compared to normal individuals [77]. Ovarian cancer is one of the most lethal forms of cancer in women, and until recently, lacked consistent biomarkers useful for its detection. Approximately 70% of cases of ovarian cancer are diagnosed in advanced stage, which has only a 20% survival rate within 5 years of diagnosis. However, if diagnosed at early stage (at Stage I), survival rate is over 90% [12]. Therefore, early diagnosis using exosomal contents may be highly important and may save large number of patients dying from ovarian cancer due to late diagnosis. Exosomes provide stable, disease-specific markers for detection, disease characterization, and disease prognosis [73]. Even though change in exosome content in circulation can be used as a diagnostic tool to detect cancer, but measuring the content of circulating exosomes is not very specific and authentic technique. However, proteomic and genetic profiling of circulating exosomes can provide better diagnosis and monitoring of therapeutic response and diagnostic tool [73].

Recent discoveries of the presence of microRNAs, epithelial cell adhesion molecule (EpCAM) and CD24 in ovarian tumor-derived exosomes have been highly promising alternatives for early detection of ovarian cancer. EpCAM is a glycoprotein that facilitates homo-typical adhesion of cells. It has been found in pseudo-stratified, transitional and simple epithelia on the basolateral surfaces and has been identified as a cargo protein in exosomes. EpCAM is expressed to a certain degree in the normal epithelia of many organs and is highly overexpressed in multiple types of carcinomas. The overexpression is correlated with an escalation of epithelial cell proliferation, as occurs in tumor development [66]. Studies by some investigators have used the magnetic activated cell sorting procedure (MACS) using magnetic micro-beads coated with EpCAM antibodies to isolate exosomes that specifically express EpCAM [12,77]. Results from these studies showed very low levels of EpCAM (0.039+0.030 mg/ml) in exosomes of normal individuals and an increase in levels related to stage and severity of ovarian carcinoma, reaching a significantly higher level of 1.42 + 0.228 mg/ml for Stage IV ovarian carcinomas. This indicates that EpCAM can be useful as a biomarker for the diagnosis of ovarian cancer as well as the detection of tumor-derived exosomes [12,66]. High EpCAM concentration is usually associated with a high concentration of CD24 [43]. CD24 is recognized as a tumor marker and is associated with poor prognosis of ovarian carcinomas. It is also expressed by many other types of malignancies as well as by some normal cell types. CD24 is a glycoprotein linked to the cell membrane via a GPI-anchor. CD24 can also be found in the cytoplasm inside MVBs and released into the extracellular environment via exosomes, in which case it is correlated with more aggressive forms of ovarian carcinoma, worsening the prognoses and therefore, shortening patients’ survival times [43].

Presently, ~2,000 microRNAs have been described in human [78] microRNAs have been reported to play important role in in cellular and developmental processes, including developmental timing, organ development, differentiation, proliferation, apoptosis and immune regulation [67]. Recently, Taylor and his colleagues reported the presence of diagnostic miRNAs from EpCAM-positive exosomes from ovarian cancer patients’ serum [12]. The discoveries for the presence of microRNAs, EpCAM, and CD24 in ovarian tumor-derived exosomes have provided promising alternatives to early detection. MicroRNAs are small non-coding RNAs ([22-25] nucleotides) that are important in the regulation of cellular processes such as differentiation, proliferation, cell death, and maturation. MicroRNAs from normal cells and tumor cells of the same tissue type have remarkably different makeup. These findings, along with the previous knowledge of the secretion of exosomes by tumor cells, have recently led to research that microRNAs may be useful as biomarkers. Ideal microRNAs used for diagnosis purpose would be absent or present at non-detectable level in the circulating exosomes of cancer-free individuals, but are aberrantly expressed in ovarian cancer and it is postulated that they may be useful in the diagnosis and prognosis of ovarian cancer. There are 8 microRNAs (miR-21, miR-141, miR-200a, miR-200b, miR-200c, miR-203, miR-205 and miR-214) that are confirmed to be diagnostic [12]. The levels of these microRNAs in serum differ slightly between the early and late stages of ovarian cancer, but differ significantly between exosomes from individuals with benign disease and those with advance stages of malignancy. Specific microRNAs have been profiled from a direct tumor tissue samples and compared to microRNAs from exosomes from the peripheral circulation. Results showed that the microRNA from tumor-derived exosomes are a correct reflection of the microRNA from the tumor, indicating that profiling the exosomal microRNA may serve as a useful tool for early diagnosis of this deadly disease instead of sampling of ovarian cancer tissues [12].

### Exosomes in immune tolerance

While some exosomes have demonstrated immune-stimulatory effects, whereas, others, such as exosomes released by intestinal epithelial cells have demonstrated the ability to induce antigen-specific tolerance. In a key study by Karlsson, these so-called “tolerosomes” were shown to carry MHC II molecules and thus assist the body in building a tolerance to food antigens [36]. Exosomes have also been reported to stimulate an immune tolerance during pregnancy [79-82].

Interestingly, in contrast to intestinal epithelial cell-derived exosomes helping to create antigen-specific tolerance, exosomes released from dendritic cells that are mounted with tumor peptides produce a specific anti-tumor response *in vitro* in human [10]. Findings by Andre and colleagues used exosomes from dendritic cells that had been pulsed with tumor peptides showed a tumor-rejection effect in patients with melanoma, prostate cancer, lymphoma, and renal cancer [33], indicating that exosomes secreted by tumor cells have the ability to stimulate an anti-tumor response by transferring tumor-specific antigens to dendritic or other antigen-presenting cells, initiating a specific immune response and thus have much potential in cancer immunotherapy [10,11]. In order for a T cell-mediated immune response to a tumor to occur, the antigen-presenting dendritic cells must be presented and then process a tumor antigen, which then may be cross-presented on MHC-I molecules to activate a T cell response, which has been demonstrated in an *in vitro* model [37].

In addition to exosomes acting as immune-stimulants, they have also demonstrated the ability to encourage tolerance in the immune system. Exosomes introduced to a patient from a donor prior to transplant surgery allow the patient longer transplant acceptance time by the immune system [71]. Studies using a rat model have shown that rat treated with bone marrow-derived exosomes prior to a heart allograft procedure showed an increase in alloantibody production, but also experienced a decreased anti-donor T cell response, and longer graft survival [83].

Another means of tumor promotion by exosomes is their capability to suppress the immune response. Experiments performed by Liu et al. [84] indicated an exosome-mediated reduction of natural killer (NK) T cells, unrelated to actions of T-regulatory cells. The same group found tumor exosomes derived from myeloid-derived suppressor cells (MDSCs) [85] and Clayton and colleagues found tumor exosomes derived from mesothelioma [85] to express (membrane-associated) Transforming Growth Factor-β (TGFβ_1_). Instead of acting as a cell cycle regulator in normal cells to induce apoptosis or cease cell proliferation, TGFβ_1_ from tumor exosomes assists in immune response evasion by tumor cells and also induces an antiproliferative effect on tumor growth and lymphocytes. The exosomal, membrane-associated form of TGFβ_1_ was more potent in its inhibitory effect than the soluble form at significantly lower doses [86].

Another proposed method of immune evasion by tumor exosomes is induction of T cell apoptosis. It is known that tumor exosomes are able to inhibit T cell proliferation. Many cancers coincide with notable amounts of Fas Ligand (FasL), which is correlated with poor prognosis [87]. FasL is a transmembrane protein in the tumor necrosis factor family. FasL is found in the membrane of exosomes that are released from tumors and it has been concluded that FasL does induce apoptosis of T cells, suppressing the immune response [16,85,88,89].

Another mechanism of immune defense involves antigen-presenting cells. Dendritic cell-derived exosomes express MHC I and II and T cell co-stimulatory molecules and deliver these complexes to dendritic cells, which are capable of activating CD4^+^ and CD8^+^ T cells and suppressing tumor growth [16,90,91] (Table 1). However, if antigen-presenting cells such as dendritic cells are not involved, tumor-derived exosomes may possess a self-promoting function. Studies have recently stressed the importance of microvesicles (exosomes) secreted by tumor cells an important means of communication between tumor cells and their environment.

### Exosomes and microvesicles

In this review we focused on exosomoes that are secreted from the inner cell membrane compartment. We did not discuss involvement of larger vesicles (100 nm-1μm) that are secreted from the cell surface membranes. Augmenting evidence indicates that both types of vesicles - smaller (exosomes) and larger (microvesicles) are secreted from the activated cells. Since ovarian cancer cells express highly tissue factor (TF) [92], they can easily activate blood platelets that are rich source of both exosomes and microvesicles. It has been demonstrated that TF-directed activation of platelets enhances in exosome/microvesicle-dependent manner metastasis of several types of cancer cells [86,93,94]. Moreover, a concept has been presented that tumor growth and expansion is directed by a network of extracellular vesicles secreted by various types of normal and malignant cells present in tumor microenvironment [95]. Its involvement in progression of ovarian cancer needs further studies.

### Exosomes and therapies

Research and clinical studies have already proven exosomes to be an accessible and reliable source of biomarkers and early diagnosis of disease, with many more avenues yet to be explored. Exosomes bode well in their role as a means of intercellular communication; they contain many different sets of molecules that have roles in various cellular processes and possess the ability to carry antigenic information. They have the advantage of being nonliving and they are easily recovered from biological fluids [15]. Perhaps the most advantageous of exosomal possibilities is their potential to serve as a key factor in finding a cure for cancer. Tumor-derived exosomes have been used to carry tumor antigens and present them to T cells, priming them to induce the anti-tumor response, resulting in tumor cell death [10,15,62]. Their ability to do this suggests that tumor-derived exosomes may be useful in developing a cell-free anti-cancer vaccine [96,97].

With the growing knowledge of exosomes functions and the inner workings of the immune response, immunotherapeutic treatments are continuously being investigated and developed. Vaccines are being created on the basis of the knowledge of antigen presenting dendritic cells that have the ability to activate CD4^+^ and CD8^+^ T cells, as well as natural killer cells. In a phase I clinical trial, dendritic cell exosomes demonstrated the ability to activate NK cell proliferation and activation, shown by an increased quantity of circulating NK cells in patients. By observing such role of exosomes, it is plausible that in near future, a cell-free anticancer vaccine will be available.

## Conclusions

Because of multiple biological and therapeutic functions of exosomes, studies on application of exosomes are moving forward with new applications of exosomes. Identification of new proteins and biomolecules in exosomes and novel mechanisms by which they communicate and deliver their functions are of great interest for diagnosis, drug delivery and therapeutic purposes.

## Competing interests

The authors declare that they have no competing interests.

## Authors’ contributions

AB prepared the first draft of the manuscript; SSK and MZR participated in its design and coordination, and wrote and revised the manuscript; HGZ provided in depth input and edited the manuscript. All authors read and approved the final manuscript.

## References

[B1] Trams EG Lauter CJ Salem N Jr Heine U Exfoliation of membrane ecto-enzymes in the form of micro-vesicles Biochim Biophys Acta 1981 645 63 70 10.1016/0005-2736(81)90512-5 6266476

[B2] Pan BT Teng K Wu C Adam M Johnstone RM Electron microscopic evidence for xternalization of the transferrin receptor in vesicular form in sheep reticulocytes J Cell Biol 1985 101 942 948 10.1083/jcb.101.3.942 2993317 PMC2113705

[B3] Johnstone RM Adam M Hammond JR Orr L Turbide C Vesicle formation during reticulocyte maturation. Association of plasma membrane activities with released vesicles (exosomes) J Biol Chem 1987 262 9412 9420 3597417

[B4] Johnstone RM Bianchini A Teng K Reticulocyte maturation and exosome release: transferrin receptor containing exosomes shows multiple plasma membrane functions Blood 1989 74 1844 1851 2790208

[B5] Harding C Heuser J Stahl P Receptor-mediated endocytosis of transferrin and recycling of the transferrin receptor in rat reticulocytes J Cell Biol 1983 97 329 39 10.1083/jcb.97.2.329 6309857 PMC2112509

[B6] Admyre C Johansson SM Qazi KR Filén JJ Lahesmaa R Norman M Neve EP Scheynius A Gabrielsson S Exosomes with immune modulatory features are present in human breast milk J Immunol 2007 179 1969 1978 17641064 10.4049/jimmunol.179.3.1969

[B7] Masyuk AI Masyuk TV Larusso NF Exosomes in the pathogenesis, diagnostics and therapeutics of liver diseases J Hepatol 2013 59 621 625 10.1016/j.jhep.2013.03.028 23557871 PMC3831338

[B8] Vella LJ Sharples RA Nisbet RM Cappai R Hill AF The role of exosomes in the processing of proteins associated with neurodegenerative diseases Eur Biophys J 2008 37 323 332 10.1007/s00249-007-0246-z 18064447

[B9] Bard MP Hegmans JP Hemmes A Luider TM Willemsen R Severijnen LA van Meerbeeck JP Burgers SA Hoogsteden HC Lambrecht BN Proteomic analysis of exosomes isolated from human malignant pleural effusions Am J Respir Cell Mol Biol 2004 31 114 21 10.1165/rcmb.2003-0238OC 14975938

[B10] Schorey JS Bhatnagar S Exosome function: from tumor immunology to pathogen biology Traffic 2008 9 871 881 10.1111/j.1600-0854.2008.00734.x 18331451 PMC3636814

[B11] Koga K Matsumoto K Akiyoshi T Kubo M Yamanaka N Tasaki A Nakashima H Nakamura M Kuroki S Tanaka M Katano M Purification, characterization and biological significance of tumor-derived exosomes Anticancer Res 2005 25 3703 3707 16302729

[B12] Taylor DD Gercel-Taylor C MicroRNA signatures of tumor-derived exosomes as diagnostic biomarkers of ovarian cancer Gynecol Oncol 2008 110 13 21 10.1016/j.ygyno.2008.04.033 18589210

[B13] Théry C Zitvogel L Amigorena S Exosomes: composition, biogenesis and function Nat Rev Immunol 2002 2 569 579 12154376 10.1038/nri855

[B14] Keller S Sanderson MP Stoeck A Altevogt P Exosomes: from biogenesis and secretion to biological function Immunol Lett 2006 107 102 108 10.1016/j.imlet.2006.09.005 17067686

[B15] van Niel G Porto-Carreiro I Simoes S Raposo G Exosomes: A Common Pathway for a Specialized Function J. Biochem 2006 140 13 21 10.1093/jb/mvj128 16877764

[B16] Théry C Regnault A Garin J Wolfers J Zitvogel L Ricciardi-Castagnoli P Raposo G Amigorena S Molecular characterization of dendritic cell-derived exosomes. Selective accumulation of the heat shock protein hsc73 J Cell Biol 1999 147 599 610 10.1083/jcb.147.3.599 10545503 PMC2151184

[B17] Simons M Raposo G Exosomes - vesicular carriers for intercellular communication Curr Opin Cell Biol 2009 21 575 81 10.1016/j.ceb.2009.03.007 19442504

[B18] Johnstone RM Ahn J A common mechanism may be involved in the selective loss of plasma membrane functions during reticulocyte maturation Biomed Biochim Acta 1990 49 S70 S75 2386531

[B19] Henderson MC Azorsa DO The genomic and proteomic content of cancer cell-derived exosomes Front Oncol 2012 2 38 22649786 10.3389/fonc.2012.00038 PMC3355967

[B20] Zech D Rana S Büchler MW Zöller M Tumor-exosomes and leukocyte activation: an ambivalent crosstalk Cell Commun Signal 2012 10 37 10.1186/1478-811X-10-37 23190502 PMC3519567

[B21] Lai RC Arslan F Lee MM Sze NS Choo A Chen TS Salto-Tellez M Timmers L Lee CN El Oakley RM Pasterkamp G de Kleijn DP Lim SK Exosome secreted by MSC reduces myocardial ischemia/reperfusion injury Stem Cell Res 2010 4 214 222 10.1016/j.scr.2009.12.003 20138817

[B22] Van Niel G Mallegol J Bevilacqua C Candalh C Brugière S Tomaskovic-Crook E Heath JK Cerf-Bensussan N Heyman M Intestinal epithelial exosomes carry MHC class II/peptides able to inform the immune system in mice Gut 2003 52 1690 1697 10.1136/gut.52.12.1690 14633944 PMC1773888

[B23] Kapsogeorgou EK Abu-Helu RF Moutsopoulos HM Manoussakis MN Salivary gland epithelial cell exosomes: A source of autoantigenic ribonucleoproteins Arthritis Rheum 2005 52 1517 1521 10.1002/art.21005 15880835

[B24] Wang G Dinkins M He Q Zhu G Poirier C Campbell A Mayer-Proschel M Bieberich E Astrocytes secrete exosomes enriched with proapoptotic ceramide and prostate apoptosis response 4 (PAR-4): potential mechanism of apoptosis induction in Alzheimer disease (AD) J Biol Chem 2012 287 21384 1395 10.1074/jbc.M112.340513 22532571 PMC3375560

[B25] Zhan R Leng X Liu X Wang X Gong J Yan L Wang L Wang Y Wang X Qian LJ Heat shock protein 70 is secreted from endothelial cells by a non-classical pathway nvolving exosomes Biochem Biophys Res Commun 2009 387 229 233 10.1016/j.bbrc.2009.06.095 19555663

[B26] Keryer-Bibens C Pioche-Durieu C Villemant C Souquère S Nishi N Hirashima M Middeldorp J Busson P Exosomes released by EBV-infected nasopharyngeal carcinoma cells convey the viral latent membrane protein 1 and the immunomodulatory protein galectin 9 BMC Cancer 2006 6 283 10.1186/1471-2407-6-283 17156439 PMC1779799

[B27] Savina A Furlán M Vidal M Colombo MI Exosome release is regulated by a calcium-dependent mechanism in K562 cells J Biol Chem 2003 278 20083 20090 10.1074/jbc.M301642200 12639953

[B28] Navabi H Croston D Hobot J Clayton A Zitvogel L Jasani B Bailey-Wood R Wilson K Tabi Z Mason MD Adams M Preparation of human ovarian cancer ascites-derived exosomes for a clinical trial Blood Cells Mol Dis 2005 35 149 152 10.1016/j.bcmd.2005.06.008 16061407

[B29] Rabinowits G Gerçel-Taylor C Day JM Taylor DD Kloecker GH Exosomal microRNA: a diagnostic marker for lung cancer Clin Lung Cancer 2009 10 1 42 46 10.3816/CLC.2009.n.006 19289371

[B30] Hessvik NP Phuyal S Brech A Sandvig K Llorente A Profiling of microRNAs in exosomes released from PC-3 prostate cancer cells Biochim Biophys Acta 1819 2012 1154 1163 10.1016/j.bbagrm.2012.08.016 22982408

[B31] Bryant RJ Pawlowski T Catto JW Marsden G Vessella RL Rhees B Kuslich C Visakorpi T Hamdy FC Changes in circulating microRNA levels associated with prostate cancer Br J Cancer 2012 106 768 774 10.1038/bjc.2011.595 22240788 PMC3322952

[B32] Takeshita N Hoshino I Mori M Akutsu Y Hanari N Yoneyama Y Ikeda N Isozaki Y Maruyama T Akanuma N Komatsu A Jitsukawa M Matsubara H Serum microRNA expression profile: miR-1246 as a novel diagnostic and prognostic biomarker for oesophageal squamous cell carcinoma Br J Cancer 2013 108 644 652 10.1038/bjc.2013.8 23361059 PMC3593570

[B33] AAndre F Schartz NE Movassagh M Flament C Pautier P Morice P Pomel C Lhomme C Escudier B Le Chevalier T Tursz T Amigorena S Raposo G Angevin E Zitvogel L Malignant effusions and immunogenic tumour-derived exosomes Lancet 2002 360 295 305 10.1016/S0140-6736(02)09552-1 12147373

[B34] Février B Vilette D Laude H Raposo G Exosomes: a bubble ride for prions? Traffic 2005 6 10 17 10.1111/j.1600-0854.2004.00247.x 15569241

[B35] Mears R Craven RA Hanrahan S Totty N Upton C Young SL Patel P Selby PJ Banks RE Proteomic analysis of melanoma-derived exosomes by two-dimensional polyacrylamide gel electrophoresis and mass spectrometry Proteomics 2004 4 4019 4031 10.1002/pmic.200400876 15478216

[B36] Karlsson M Lundin S Dahlgren U Kahu H Pettersson I Telemo E "Tolerosomes" are produced by intestinal epithelial cells Eur J Immunol 2001 31 2892 2900 10.1002/1521-4141(2001010)31:10<2892::AID-IMMU2892>3.0.CO;2-I 11592064

[B37] Wolfers J Lozier A Raposo G Regnault A Théry C Masurier C Flament C Pouzieux S Faure F Tursz T Angevin E Amigorena S Zitvogel L Tumor-derived exosomes are a source of shared tumor rejection antigens for CTL cross-priming Nat Med 2001 7 297 303 10.1038/85438 11231627

[B38] Keller S Rupp C Stoeck A Runz S Fogel M Lugert S Hager HD Abdel-Bakky MS Gutwein P Altevogt P CD24 is a marker of exosomes secreted into urine and amniotic fluid Kidney Int 2007 72 1095 1102 10.1038/sj.ki.5002486 17700640

[B39] Li QL Bu N Yu YC Hua W Xin XY Exvivo experiments of human ovarian cancer ascites-derived exosomes presented by dendritic cells derived from umbilical cord blood for immunotherapy treatment Clin Med Oncol 2008 2 461 467 21892318 10.4137/cmo.s776 PMC3161644

[B40] Almqvist N Lönnqvist A Hultkrantz S Rask C Telemo E Serum-derived exosomes from antigen-fed mice prevent allergic sensitization in a model of allergic asthma Immunology 2008 125 21 27 10.1111/j.1365-2567.2008.02812.x 18355242 PMC2526256

[B41] Gallo A Tandon M Alevizos I Illei GG The majority of microRNAs detectable in serum and saliva is concentrated in exosomes PLoS One 2012 7 e30679 10.1371/journal.pone.0030679 22427800 PMC3302865

[B42] Ogawa Y Kanai-Azuma M Akimoto Y Kawakami H Yanoshita R Exosome-like vesicles with dipeptidyl peptidase IV in human saliva Biol Pharm Bull 2008 31 6 1059 1062 10.1248/bpb.31.1059 18520029

[B43] Runz S Keller S Rupp C Stoeck A Issa Y Koensgen D Mustea A Sehouli J Kristiansen G Altevogt P Malignant ascites-derived exosomes of ovarian carcinoma patients contain CD24 and EpCAM Gynecol Oncol 2007 107 563 571 10.1016/j.ygyno.2007.08.064 17900673

[B44] Saman S Kim W Raya M Visnick Y Miro S Saman S Jackson B McKee AC Alvarez VE Lee NC Hall GF Exosome-associated tau is secreted in tauopathy models and is selectively phosphorylated in cerebrospinal fluid in early Alzheimer disease J Biol Chem 2012 287 3842 3849 10.1074/jbc.M111.277061 22057275 PMC3281682

[B45] Street JM Barran PE Mackay CL Weidt S Balmforth C Walsh TS Chalmers RT Webb DJ Dear JW Identification and proteomic profiling of exosomes in human cerebrospinal fluid J Transl Med 2012 10 5 10.1186/1479-5876-10-5 22221959 PMC3275480

[B46] Qiu S Duan X Geng X Xie J Gao H Antigen-specific activities of CD8+ T cells in the nasal mucosa of patients with nasal allergy Asian Pac J Allergy Immunol 2012 30 107 13 22830289

[B47] Logozzi M De Milito A Lugini L Borghi M Calabrò L Spada M Perdicchio M Marino ML Federici C Iessi E Brambilla D Venturi G Lozupone F Santinami M Huber V Maio M Rivoltini L Fais S High levels of exosomes expressing CD63 and caveolin-1 in plasma of melanoma patients PLoS One 2009 4 e5219 10.1371/journal.pone.0005219 19381331 PMC2667632

[B48] Record M Subra C Silvente-Poirot S Poirot M Exosomes as ntercellular signalosomes and pharmacological effectors Biochem Pharmacol 2011 81 1171 1182 10.1016/j.bcp.2011.02.011 21371441

[B49] Record M Zhang H-G Exosomal Lipids in Cell-Cell Communication Emerging Concepts of Tumor Exosome-Mediated Cell-Cell Communication 2013 New York, NY USA: Springer 47 68

[B50] Ostrowski M Carmo NB Krumeich S Fanget I Raposo G Savina A Moita CF Schauer K Hume AN Freitas RP Goud B Benaroch P Hacohen N Fukuda M Desnos C Seabra MC Darchen F Amigorena S Moita LF Thery C Rab27a and Rab27b control different steps of the exosome secretion pathway Nat Cell Biol 2010 12 1–13 19 30 19966785 10.1038/ncb2000

[B51] Hsu C Morohashi Y Yoshimura S Manrique-Hoyos N Jung S Lauterbach MA Bakhti M Grønborg M Möbius W Rhee J Barr FA Simons M Regulation of exosome secretion by Rab35 and its GTPase-activating proteins TBC1D10A-C J Cell Biol 2010 189 223 32 10.1083/jcb.200911018 20404108 PMC2856897

[B52] Savina A Vidal M Colombo MI The exosome pathway in K562 cells is regulated by Rab11 J Cell Sci 2002 115 2505 2515 12045221 10.1242/jcs.115.12.2505

[B53] Yu X Harris SL Levine AJ The regulation of exosome secretion: a novel function of the p53 protein Cancer Res 2006 66 4795 4801 10.1158/0008-5472.CAN-05-4579 16651434

[B54] Yu XT Riley T Levine AJ The regulation of the endosomal compartment by p53 the tumor suppressor gene FEBS J 2009 276 2201 2212 10.1111/j.1742-4658.2009.06949.x 19302216

[B55] Azmi AS Bao B Sarkar FH Exosomes in cancer development, metastasis, and drug resistance: a comprehensive review Cancer Metastasis Rev 2013 Epub ahead of print 10.1007/s10555-013-9441-9 PMC3843988 23709120

[B56] Parolini I Federici C Raggi C Lugini L Palleschi S De Milito A Coscia C Iessi E Logozzi M Molinari A Colone M Tatti M Sargiacomo M Fais S Microenvironmental pH is a key factor for exosome traffic in tumor cells J Biol Chem 2009 284 34211 34222 10.1074/jbc.M109.041152 19801663 PMC2797191

[B57] Stoorvogel W Kleijmeer MJ Geuze HJ Raposo G The biogenesis and functions of exosomes Traffic 2002 3 321 330 10.1034/j.1600-0854.2002.30502.x 11967126

[B58] Keller S König AK Marmé F Runz S Wolterink S Koensgen D Mustea A Sehouli J Altevogt P Systemic presence and tumor-growth promoting effect of ovarian carcinoma released exosomes Cancer Lett 2009 278 73 81 10.1016/j.canlet.2008.12.028 19188015

[B59] Mathivanan S Fahner CJ Reid GE Simpson RJ ExoCarta 2012: database of exosomal proteins, RNA and lipids Nucleic Acids Res 2012 40 D1241 D1244 10.1093/nar/gkr828 21989406 PMC3245025

[B60] Mathivanan S Simpson RJ ExoCarta: A compendium of exosomal proteins and RNA Proteomics 2009 9 4997 5000 10.1002/pmic.200900351 19810033

[B61] Lamparski HG Metha-Damani A Yao JY Patel S Hsu DH Ruegg C Le Pecq JB Production and characterization of clinical grade exosomes derived from dendritic cells J Immunol Methods 2002 270 211 226 10.1016/S0022-1759(02)00330-7 12379326

[B62] Cho JA Park H Lim EH Lee KW Exosomes: a new delivery system for tumor antigens in cancer immunotherapy Int J Cancer 2005 114 613 622 10.1002/ijc.20757 15609328

[B63] Hegmans JP Bard MP Hemmes A Luider TM Kleijmeer MJ Prins JB Zitvogel L Burgers SA Hoogsteden HC Lambrecht BN Proteomic analysis of exosomes secreted by human mesothelioma cells Am J Pathol 2004 164 1807 1815 10.1016/S0002-9440(10)63739-X 15111327 PMC1615654

[B64] Valadi H Ekström K Bossios A Sjöstrand M Lee JJ Lötvall JO Exosome-mediated transfer of mRNAs and microRNAs is a novel mechanism of genetic exchange between cells Nat Cell Biol 2007 9 654 659 10.1038/ncb1596 17486113

[B65] Taylor DD Zacharias W Gercel-Taylor C Exosome isolation for proteomic analyses and RNA profiling Methods Mol Biol 2011 728 235 246 10.1007/978-1-61779-068-3_15 21468952

[B66] Lim LP Lau NC Garrett-Engele P Grimson A Schelter JM Castle J Bartel DP Linsley PS Johnson JM Microarray analysis shows that some microRNAs downregulate large numbers of target mRNAs Nature 2005 433 769 773 10.1038/nature03315 15685193

[B67] Taft RJ Pang KC Mercer TR Dinger M Mattick JS Non-coding RNAs: regulators of disease J Pathol 2010 220 126 139 10.1002/path.2638 19882673

[B68] Hong BS Cho JH Kim H Choi EJ Rho S Kim J Kim JH Choi DS Kim YK Hwang D Gho YS Colorectal cancer cell-derived microvesicles are enriched in cell cycle-related mRNAs that promote proliferation of endothelial cells BMC Genomics 2009 10 556 10.1186/1471-2164-10-556 19930720 PMC2788585

[B69] Hannafon BN Ding WQ Intercellular Communication by Exosome-Derived microRNAs in Cancer Int J Mol Sci 2013 14 14240 14269 10.3390/ijms140714240 23839094 PMC3742242

[B70] Ratajczak J Miekus K Kucia M Zhang J Reca R Dvorak P Ratajczak MZ Embryonic stem cell-derived microvesicles reprogram hematopoietic progenitors: evidence for horizontal transfer of mRNA and protein delivery Leukemia 2006 20 847 56 10.1038/sj.leu.2404132 16453000

[B71] Fevrier B Raposo G Exosomes: endosomal-derived vesicles shipping extracellular messages Curr Opin Cell Biol 2004 16 415 421 10.1016/j.ceb.2004.06.003 15261674

[B72] Taylor DD Gercel-Taylor C Exosomes/microvesicles: mediators of cancer-associated immunosuppressive microenvironments Semin Immunopathol 2011 33 441 54 10.1007/s00281-010-0234-8 21688197

[B73] Liang B Peng P Chen S Li L Zhang M Cao D Yang J Li H Gui T Li X Shen K Characterization and proteomic analysis of ovarian cancer-derived exosomes Proteomics 2013 80C 171 182 23333927 10.1016/j.jprot.2012.12.029

[B74] Taylor DD Gerçel-Taylor C Tumour-derived exosomes and their role in cancer-associated T-cell signalling defects Br J Cancer 2005 92 305 311 15655551 10.1038/sj.bjc.6602316 PMC2361848

[B75] Peng P Yan Y Keng S Exosomes in the ascites of ovarian cancer patients: origin and effects on anti-tumor immunity Oncol Rep 2011 25 749 762 21181093 10.3892/or.2010.1119

[B76] Taylor DD Gercel-Taylor C The origin, function, and diagnostic potential of RNA within extracellular vesicles present in human biological fluids Front Genet 2013 4 142 23908664 10.3389/fgene.2013.00142 PMC3726994

[B77] Went PT Lugli A Meier S Bundi M Mirlacher M Sauter G Dirnhofer S Frequent EpCam protein expression in human carcinomas Hum Pathol 2004 35 122 128 10.1016/j.humpath.2003.08.026 14745734

[B78] Oliveira LJ Barreto RS Perecin F Mansouri-Attia N Pereira FT Meirelles FV Modulation of maternal immune system during pregnancy in the cow Reprod Domest Anim 2012 47 Suppl 4 384 393 22827396 10.1111/j.1439-0531.2012.02102.x

[B79] Delorme-Axford E Donker RB Mouillet JF Chu T Bayer A Ouyang Y Wang T Stolz DB Sarkar SN Morelli AE Sadovsky Y Coyne CB Human placental trophoblasts confer viral resistance to recipient cells Proc Natl Acad Sci USA 2013 110 12048 12053 10.1073/pnas.1304718110 23818581 PMC3718097

[B80] Williams JL Gatson NN Smith KM Almad A McTigue DM Whitacre CC Serum exosomes in pregnancy-associated immune modulation and neuroprotection during CNS autoimmunity Clin Immunol 2013 149 236 243 10.1016/j.clim.2013.04.005 23706172 PMC3778091

[B81] Taylor DD Akyol S Gercel-Taylor C Pregnancy-associated exosomes and their modulation of T cell signaling J Immunol 2006 176 1534 1542 16424182 10.4049/jimmunol.176.3.1534

[B82] Pêche H Heslan M Usal C Amigorena S Cuturi MC Presentation of donor major histocompatibility complex antigens by bone marrow dendritic cell-derived exosomes modulates allograft rejection Transplantation 2003 76 1503 1510 10.1097/01.TP.0000092494.75313.38 14657694

[B83] Liu C Yu S Zinn K Wang J Zhang L Jia Y Kappes JC Barnes S Kimberly RP Grizzle WE Zhang HG Murine mammary carcinoma exosomes promote tumor growth by suppression of NK cell function J Immunol 2006 176 1375 1385 16424164 10.4049/jimmunol.176.3.1375

[B84] Xiang X Liu Y Zhuang X Zhang S Michalek S Taylor DD Grizzle W Zhang HG TLR2-mediated expansion of MDSCs is dependent on the source of tumor exosomes Am J Pathol 2010 177 1606 1610 10.2353/ajpath.2010.100245 20802178 PMC2947257

[B85] Clayton A Mitchell JP Court J Mason MD Tabi Z Human tumor-derived exosomes selectively impair lymphocyte responses to interleukin-2 Cancer Res 2007 67 7458 7466 10.1158/0008-5472.CAN-06-3456 17671216

[B86] Janowska-Wieczorek A Marquez-Curtis LA Wysoczynski M Ratajczak MZ Enhancing effect of platelet-derived microvesicles on the invasive potential of breast cancer cells Transfusion 2006 46 1199 209 10.1111/j.1537-2995.2006.00871.x 16836568

[B87] Abusamra AJ Zhong Z Zheng X Li M Ichim TE Chin JL Min WP Tumor exosomes expressing Fas ligand mediate CD8+ T-cell apoptosis Blood Cells Mol Dis 2005 35 169 173 10.1016/j.bcmd.2005.07.001 16081306

[B88] Viaud S Terme M Flament C Taieb J André F Novault S Escudier B Robert C Caillat-Zucman S Tursz T Zitvogel L Chaput N Dendritic cell-derived exosomes promote natural killer cell activation and proliferation: a role for NKG2D ligands and IL-15Ralpha PLoS One 2009 4 e4942 10.1371/journal.pone.0004942 19319200 PMC2657211

[B89] Morse MA Garst J Osada T Khan S Hobeika A Clay TM Valente N Shreeniwas R Sutton MA Delcayre A Hsu DH Le Pecq JB Lyerly HK A phase I study of dexosome immunotherapy in patients with advanced non-small cell lung cancer J Transl Med 2005 3 9 10.1186/1479-5876-3-9 15723705 PMC551593

[B90] Admyre C Grunewald J Thyberg J Gripenbäck S Tornling G Eklund A Scheynius A Gabrielsson S Exosomes with major histocompatibility complex class II and co-stimulatory molecules are present in human BAL fluid Eur Respir J 2003 22 578 583 10.1183/09031936.03.00041703 14582906

[B91] Abu Saadeh F Norris L O'Toole S Mohamed BM Langhe R O'Leary J Gleeson N Tumour expresion of tissue factor and tissue factor pathway inhibitor in ovarian cancer- relationship with venous thrombosis risk Thromb Res 2013 132 627 634 10.1016/j.thromres.2013.09.016 24094893

[B92] Janowska-Wieczorek A Wysoczynski M Kijowski J Marquez-Curtis L Machalinski B Ratajczak J Ratajczak MZ Microvesicles derived from activated platelets induce metastasis and angiogenesis in lung cancer Int J Cancer 2005 113 752 60 10.1002/ijc.20657 15499615

[B93] Wysoczynski M Liu R Kucia M Drukala J Ratajczak MZ Thrombin regulates the metastatic potential of human rhabdomyosarcoma cells: distinct role of PAR1 and PAR3 signaling Mol Cancer Res 2010 8 677 90 10.1158/1541-7786.MCR-10-0019 20442298 PMC2896479

[B94] Wysoczynski M Ratajczak MZ Lung cancer secreted microvesicles: underappreciated modulators of microenvironment in expanding tumors Int J Cancer 2009 125 1595 603 10.1002/ijc.24479 19462451 PMC2769262

[B95] Wang B Zhuang X Deng ZB Jiang H Mu J Wang Q Xiang X Guo H Zhang L Dryden G Yan J Miller D Zhang HG Targeted drug delivery to intestinal macrophages by bioactive nanovesicles released from grapefruit Mol Ther 2013 10.1038/mt.2013.190 PMC3944329 23939022

[B96] Ju S Mu J Dokland T Zhuang X Wang Q Jiang H Xiang X Deng ZB Wang B Zhang L Roth M Welti R Mobley J Jun Y Miller D Zhang HG Grape exosome-like nanoparticles induce intestinal stem cells and protect mice from DSS-induced colitis Mol Ther 2013 21 1345 1357 10.1038/mt.2013.64 23752315 PMC3702113

[B97] Wang Q Zhuang X Mu J Deng ZB Jiang H Zhang L Xiang X Wang B Yan J Miller D Zhang HG Delivery of therapeutic agents by nanoparticles made of grapefruit-derived lipids Nat Commun 1867 2013 4 10.1038/ncomms2886 PMC4396627 23695661

